# Growth of Carbon Nanofibers and Carbon Nanotubes by Chemical Vapour Deposition on Half-Heusler Alloys: A Computationally Driven Experimental Investigation

**DOI:** 10.3390/ma17133144

**Published:** 2024-06-27

**Authors:** Ioannis G. Aviziotis, Apostolia Manasi, Afroditi Ntziouni, Georgios P. Gakis, Aikaterini-Flora A. Trompeta, Xiaoying Li, Hanshan Dong, Costas A. Charitidis

**Affiliations:** 1 Research Lab of Advanced, Composite, Nano-Materials and Nanotechnology, Materials Science and Engineering Department, School of Chemical Engineering, National Technical University of Athens, 9 Heroon Polytechneiou Street, 15780 Athens, Greece; javiziot@chemeng.ntua.gr (I.G.A.); gakisg@chemeng.ntua.gr (G.P.G.); ktrompeta@chemeng.ntua.gr (A.-F.A.T.); 2 School of Metallurgy and Materials, University of Birmingham, Birmingham B15 2TT, UK; x.li.1@bham.ac.uk (X.L.); h.dong.20@bham.ac.uk (H.D.)

**Keywords:** carbon nanofibers, carbon nanotubes, catalytic chemical vapour deposition, computationally designed experiments, half-Heusler alloys, thermoelectric materials

## Abstract

The possibility of directly growing carbon nanofibers (CNFs) and carbon nanotubes (CNTs) on half-Heusler alloys by Chemical Vapour Deposition (CVD) is investigated for the first time, without using additional catalysts, since the half-Heusler alloys per se may function as catalytic substrates, according to the findings of the current study. As a carbon source, acetylene is used in the temperature range of 700–750 °C. The n-type half-Heusler compound ${\left({Zr}_{0.4}{Ti}_{0.6}\right)}_{0.33}{Ni}_{0.33}{\left({Sn}_{0.98}{Sb}_{0.02}\right)}_{0.33}$ is utilized as the catalytic substrate. At first, a computational model is developed for the CVD reactor, aiming to optimize the experimental process design and setup. The experimental process conditions are simulated to investigate the reactive species concentrations within the reactor chamber and the activation of certain reactions. SEM analysis confirms the growth of CNFs with diameters ranging from 450 nm to 1 μm. Raman spectroscopy implies that the formed carbon structures resemble CNFs rather than CNTs, and that amorphous carbon also co-exists in the deposited samples. From the characterization results, it may be concluded that a short reaction time and a low acetylene flow rate lead to the formation of a uniform CNF coating on the surface of half-Heusler alloys. The purpose of depositing carbon nanostructures onto half-Heusler alloys is to improve the current transfer, generated from these thermoelectric compounds, by forming a conductive coating on their surface.

## 1. Introduction

The potential of carbon nanofibers (CNFs) and carbon nanotubes (CNTs) with respect to their thermomechanical properties is far from being exhausted in today’s scientific community, since the highly complex mechanisms of structure formation in fibre production and the process parameter–structure–property relationship have only been explored in the academic sector [1]. With the targeted control of their properties and their combination with other materials, completely new applications are conceivable in addition to the significant improvement in existing materials for a range of applications. These include, amongst others, new crash-absorbing structures in automotive engineering [2,3], more efficient gas diffusion structures for fuel cells [4,5,6], adsorptive fibres for hydrogen high-pressure tanks, next-generation energy storage systems, cell-compatible fibres to replace nerve tracts in paraplegia, and prosthetics with tailored properties to avoid stress shielding.

Heusler and half-Heusler alloys (X_2_YZ and XYZ, respectively) are special categories of intermetallic compounds which typically consist of transition metals in groups 8–12 for X, 3–8 for Y, and typical metals in group 13–15 for Z [7]. Heusler alloys have great features and they have been popular as magnetic (spintronic), thermoelectric, and shape memory materials [8,9,10,11,12]. Only recently have they also been investigated for their catalytic properties mainly in hydrogenation and oxidation reactions [13]. One interesting feature of Heusler alloys is that their electronic structure can be tailored by elemental substitution in accordance with the rigid-band approximation [13,14]. This enables the tuning of the functional properties. Heusler alloys are potentially valuable catalysts because there are so many possible sets of elements, and electronic tuning can be performed by elemental substitution. There are recent literature reports where half-Heusler alloys are tested as catalysts in the hydrogenation of propyne [15,16], in the oxidation of carbon monoxide [15], in the steam reforming of methanol [14], in ammonia decomposition [17], and in theoretical studies where their adsorption properties are investigated [18].

In this work, half-Heusler alloys have been used as catalytic substrates for the growth of CNF and CNT coatings, through Chemical Vapour Deposition (CVD). To the best of the authors’ knowledge, this is the first time that half-Heusler alloys have been applied for such a catalytic process with the exception of a literature report where the authors observed the unintentional CNF formation on the surface of the CO_2_TiAl half-Heusler due to substrate poisoning during methanol steam reforming [14]. For the experimental procedure, a CVD reactor was used which has been previously described in detail [19].

The developed process has the ultimate goal of improving the electrical contact of the half-Heusler alloys in energy harvesting applications, which will be a topic for a future publication. However, at the same time, we take advantage of the catalytic properties of the half-Heusler alloys to grow the targeted CNFs and CNTs without the use of an external catalyst source. Due to the novelty of the process, a computational, macroscopic model is developed to assist in the design of experiments in terms of flow and temperature conditions in the CVD reactor. The framework used for model development is based on previous works concerning the modeling of CVD reactors for the production of CNTs on established catalysts, and it has been validated by experimental measurements [20,21].

## 2. Materials and Methods

### 2.1. Experimental Section

#### 2.1.1. Half-Heusler Alloys

The half-Heusler specimens used in this study were provided by MBN Νanomaterialia, Italy. The synthesis of the n-type half Heusler compound was conducted on ΜΒΝ Nanomaterialia’s industrial plants, utilizing a piece of patented specific mechanical alloying equipment based on oscillating high-energy ball mills in which the grinding means are accelerated to impact working surfaces at high velocities in an ordered manner. In this way, the kinetic energy from grinding is purely transferred during the impact to the powders, limiting abrasion and shear effects that normally lead to contamination from the milling means to the milled material. Industrial-grade metal powder of Zr, Ti, Ni, Sn, and Sb was used in the right proportions to obtain the target composition ${\left({Zr}_{0.4}{Ti}_{0.6}\right)}_{0.33}{Ni}_{0.33}{\left({Sn}_{0.98}{Sb}_{0.02}\right)}_{0.33}$, and the mechanical alloying process was conducted in an inert Ar atmosphere. The powder was kept in the inert atmosphere also during sieving, which was performed to remove particles above 100 µm for a better consolidation. The powder material was then consolidated into pellets via hot pressing using the current-assisted sintering press Dr. Fritsch DSP 475, operated at 800 °C and 50 MPa for 4 min. The formation of the desired crystal phase was confirmed by XRD, acquired on a Rigaku MiniFlex600, Neu-Isenburg, Germany, using Co Kα radiation.

#### 2.1.2. CVD Process Description

The CVD reactor used for the experimental procedure consists of an electrically heated furnace to heat the reactor with three temperature-controlled heating zones to provide better control of the temperature. At the centre of the 1 m length, 3.4 cm diameter quartz tube, a silicon wafer was placed to hold the half-Heusler samples. The used half-Heusler compounds were of n-type with the chemical formula ${\left({Zr}_{0.4}{Ti}_{0.6}\right)}_{0.33}{Ni}_{0.33}{\left({Sn}_{0.98}{Sb}_{0.02}\right)}_{0.33}$, and they were provided for the CVD experiments in the form of compact samples with dimensions of 1 cm × 1 cm × 5 mm. The inlet system consists of a metallic-tube network for feeding the required gasses, namely acetylene and nitrogen, into the reactor, whereas the outlet system is connected to an ethanol-filled scrubber to capture the gases, particles and organic by-products.

For the experimental procedure, various operating conditions were tested according to the directions provided by the computational model and simulations, since there is no established protocol for the deposition of CNFs/CNTs on half-Heusler alloys. A summary of those conditions is presented in Table 1, where HH denotes the half-Heusler compound. The flow rate of C_2_H_2_ was equal to 46.4 mL/min for all cases, while the N_2_ flow varied, in order to change the total concentration of the C_2_H_2_ in the gas mixture. The reaction time was also set to half for the last two experiments. The reaction temperature was set to 700 °C and a trial was also made at 750 °C. In one of the cases, H_2_ was also inserted into the reaction chamber, to see if it will favour the reaction. Last but not least, thermal annealing took place for the HH4 experiment to improve the quality of the produced nanofibers.

#### 2.1.3. Characterization of Produced Coatings

Initial scanning electron microscopy (SEM) characterization was conducted in a tabletop SEM (15–30,000×, @5 kV, 15 kV, TM3030, Hitachi High Technologies, Tokyo, Japan), equipped with an EDS Bruker Quantax 70 Energy-Dispersive X-ray Spectrophotometer, available in R-NanoLab, at the School of Chemical Engineering, National Technical University of Athens, Greece. High-resolution SEM analysis was conducted by using the Thermo Fisher Scientific Company’s Apreo 2, Waltham, MA, USA, equipped with an EDS for elemental analysis, located at the School of Metallurgy and Materials, University of Birmingham, UK. An in situ measurement of the carbon fibre diameters was conducted during the SEM observation by compensating the sample working distance and tilting angles.

Raman analyses were conducted with a Renishaw inVia™, Ann Arbor, MI, USA, Reflex micro-Raman system equipped with a 532 nm laser, a diffraction grating grid of 1800 L/mm, and a confocality of 65 µm using a ×20 objective and 5% laser power, available at the School of Chemical Engineering, National Technical University of Athens, Greece.

### 2.2. Computational Section

A three-dimensional, Computational Fluid Dynamics (CFD), macroscopic model was developed for the experimental CVD reactor, aiming to inform the experimental process design and setup. In particular, the experimental process conditions were simulated in order to investigate the reactive species concentrations within the reactor chamber, and the activation of certain reactions. In this way, important information can be provided for the experimental process design. Furthermore, the model results can assist the interpretation of experimental findings, by providing insight regarding the various phenomena and mechanisms that take place within the reactor, which are not easily accessible by experimental measurements.

Although a detailed model should include the surface mechanisms taking place on the catalyst surface, the present model only deals with the phenomena taking place in the gas phase, within the reactor chamber. This is due to the fact that the catalyst used in the present work for the production of CNFs/CNTs is novel and unexplored for the particular process; thus, the kinetics and activation energies of the different reaction steps are unknown. However, as it has been shown in previous works where the theoretical predictions are compared to experimental measurements, such a model is valid and provides invaluable information for the design of experimental processes [11,12]. Therefore, the present model was developed using the same principles, without taking into account the surface mechanisms. The governing equations of the CFD model and the main mechanisms taken into account for the present study are presented below.

#### 2.2.1. Governing Equations and Model Assumptions

The CFD model consists of the governing equations for the conservation of mass, momentum, and energy, coupled with the conservation of chemical species. Such equations have been extensively used for the modelling of similar deposition processes [22,23,24].

The gas flow inside the CVD reactor was modelled as a continuum medium, assuming laminar flow which was validated using the Reynolds and Knudsen numbers (Re = 11.9, Kn = 9.54 × 10^−6^). The gas mixture parameters, such as thermal conductivity, dynamic viscosity, and diffusion coefficients for the chemical species, were calculated using the kinetic gas theory [25]. The Lennard–Jones parameters for the species were obtained from the CHEMKIN-PRO database [26]. Adequate boundary conditions were also imposed on the corresponding boundaries to simulate the experimental conditions. In particular, a no-slip condition was imposed on the reactor walls, the outlet pressure was set to atmospheric, while the N_2_ and acetylene flow rates were set to the corresponding experimental flow rates. The wall temperature within the heating zone was set to the oven temperature, while the inlet temperature was ambient. The reactor walls outside the heating zone exchange heat with the environment with an expression given by the following equation:

(1) $$Q=h\times (T-T_{ext}),$$

where h is the heat transfer coefficient and *T_ext_* is the ambient temperature.

The described set of equations was discretized and solved using Comsol Multiphysics^®^, Burlington, MA, USA, with the finite element method. A quadratic basis function was selected for the velocity, while a linear basis function set was used for temperature, chemical species, and pressure. The computational domain was discretized with a computational mesh built in Comsol Multiphysics v5.5. The mesh consisted of 68,366 tetrahedral elements. The final mesh was selected after a dependence study regarding the effect of the computational mesh on the model solution.

#### 2.2.2. Gas-Phase Reactions

Besides the transport phenomena within the reactor chamber, the model also takes into account the chemical reactions occurring in the gas phase. As previously reported, acetylene in the gas mixture can react and decompose through pyrolysis during CVD, once entering the heating zone of the reactor [27]. The acetylene pyrolysis consists of a chain of numerous reactions that have been previously reported in detail [27,28,29]. In summary, the acetylene decomposition is initiated by the reaction [29]:

C_2_H_2_ + C_2_H_2_ → C_4_H_3_ +H (2)

This is also the energetically limiting step, with an activation energy of 2.62 eV [25]. The initial hydrogen atom is released and subsequently reacts with gas-phase hydrocarbons:

C_2_H_2_ +H → C_2_H_3_ (3)

C_2_H_3_ + C_2_H_2_ → C_4_H_4_ +H (4)

This chain of reactions leads to the formation of polycyclic aromatic hydrocarbon (PAH) molecules, which may result in the formation of soot and carbon impurities [26,27]. These reactions have a lower activation energy, compared to R1 [24]. Furthermore, the direct dissociation of acetylene is described [24]:

C_2_H_2_ → C_2_H + H (5)

However, reaction 5 has a high activation energy of 4.64 eV [29], which renders it improbable to occur in the investigated temperature range of the present study, so it was not taken into account.

Therefore, as in previous works, the chain of reactions that take place during acetylene decomposition was lumped into a single step in order to save computational time [11,12]. The gas-phase reaction used in the present model is given by the following equation:

C_2_H_2_ + C_2_H_2_ → C_4_H_4_ (6)

where C_4_H_4_ is a pseudo-species representing the hydrocarbons formed during acetylene gas-phase reactions. It contributes only in the formation of carbon impurities. The reaction rate was assumed to follow first-order Arrhenius kinetics, given by the following expression:

(7) $$R_{gas}=A_{f}\cdot e^{\left(\frac{-E_{a,gas}}{RT}\right)}{\cdot \;C}_{acet}^{2}$$

where *C_acet_* is the acetylene gas-phase concentration, A_f_ is the pre-exponential factor, taken from [20,21], and E_a,gas_ is the activation energy, taken as the activation energy of the energetically limiting step of the acetylene pyrolysis [29]. The gas-phase reaction rate of Equation (7) is imposed as a source term to the acetylene species conservation equation.

## 3. Results

### 3.1. Characterization of the Catalytic Substrate

#### 3.1.1. Elemental Analysis through SEM/EDS

**Figure 1:**
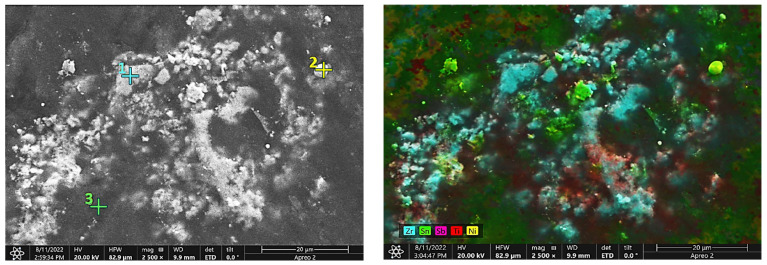
SEM/EDS analysis on the surface of the half-Heulser alloy on three different spots, marked with coloured crosses.

The surface of the half-Heusler substrate was characterized through SEM/EDS mapping (Figure 1) and the elemental composition of three different spots, as marked with coloured crosses, is presented in Table 2. As it is observed, not all local regions consist of the same elements. There are differences in the composition from spot to spot, which does not affect the thermoelectric performance of the material, which is a bulk property, but it may affect the deposition of the carbon nanostructures. Specifically, spot No. 1 is rich in Zr, spot No. 2 in Ni, and spot No. 3 in Sn. Regions that are rich in Ni will potentially favour the CVD reaction and, in turn, the growth of CNFs/CNTs.

On the other hand, it is clear from the elemental composition analysis presented in Table 2 that there are regions of the samples already containing carbon. This carbon comes from the sintering process, due to the graphite moulds that are used, and it might have an effect during the CVD process since it can form carbon structures due to the applied temperature. Unfortunately, there is no way to realize whether this carbon influences the deposition process; however, since the deposition of CNFs is homogeneous as it is shown in the following, it is safe to assume that its effect on the CVD process is minimal.

#### 3.1.2. Crystallographic Structure of Half-Heusler Alloy

**Figure 2:**
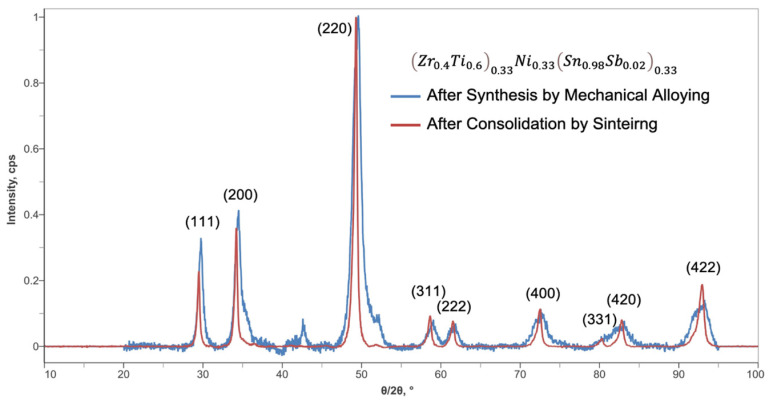
The XRD pattern of the ${\left({Zr}_{0.4}{Ti}_{0.6}\right)}_{0.33}{Ni}_{0.33}{\left({Sn}_{0.98}{Sb}_{0.02}\right)}_{0.33}$ half-Heusler alloy prior to and after sintering.

The high-energy ball milling process can lead to different materials while starting from the same initial composition, depending on the chosen process conditions. For this reason, to monitor the evolution of synthesis (i.e., presence or absence of desired phases) and to optimize the process parameters (i.e., time, rotation speed), the XRD technique is used. XRD analysis is conducted both on treated powder and sintered samples to monitor the reactivity of materials and to control the properties of the final products, as it can be seen in Figure 2, where no severe differences are observed prior to and after the sintering of the half-Heusler alloy. However, what is observed in the XRD spectrum of Figure 2 is that there is a shift towards lower angles after the consolidation by sintering, indicating the induction of structural strain within the crystal lattice of the half-Heusler compound. This effect has also been observed in the literature in other compound types such as perovskites with the induction of chemical tensile strain in the crystal lattice, and it is possible to calculate it with the Williamson–Hall analysis [30]. However, such an analysis is out of the scope of this work since the purpose is to investigate the possibility of depositing carbon structures on half-Heusler compounds.

### 3.2. Numerical Analysis

#### 3.2.1. Flow and Temperature Field

**Figure 3:**
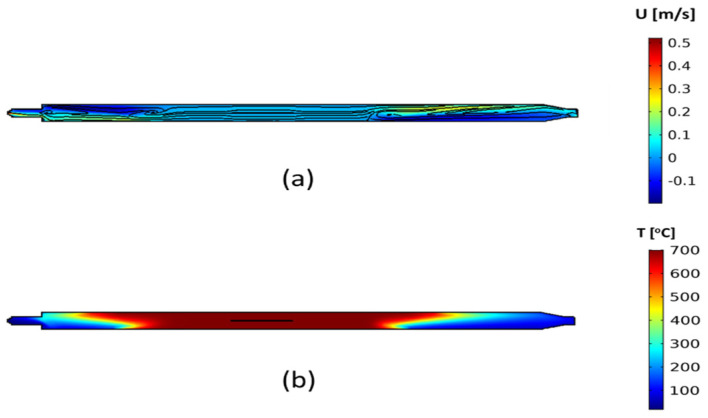
(**a**) The velocity vector and streamlines and (**b**) the temperature distribution within the CVD reactor, for the simulated case using the conditions of HH1.

The first step of the analysis consists of the flow and temperature field within the CVD reactor. Representative results for the flow and temperature field are presented in Figure 3, using the conditions of HH1, which shows that gas flow recirculation occurs at the heating zone inlet and outlet (Figure 3a). The gas mixture has a plug flow near the reactor inlet. However, due to the rapid heating as the mixture enters the heated zone of the reactor (Figure 3b), gas flow recirculation arises towards the reactor inlet. A similar behaviour is observed upon the exit of the gas from the furnace, due to the sharp temperature gradient. This can be seen in Figure 3a, where the velocity vector (x dimension velocity, where x is the dimension along the reactor length) shows both positive and negative values at the regions close to the entrance and exit of the furnace. Close to the inlet, the gas mixture flows close to the lower tube wall (positive velocity), while some gas flows back in the opposite direction, close to the upper reactor wall (negative velocity). These phenomena are attributed to buoyancy effects due to the rapid gas heating, upon entering the heating zone [31,32,33]. This influences the temperature distribution close to the inlet of the reactor (Figure 3b), which in turn may result in unwanted gas-phase reactions outside of the reactor heated zone. This may activate gas-phase reactions (acetylene pyrolysis), which are unwanted, especially in non-heated zones of the reactor, as the reactor byproducts, such as PAHs, can condense on the reactor unheated walls, causing equipment damage.

#### 3.2.2. Chemical Species

##### Effect of Process Temperature

**Figure 4:**
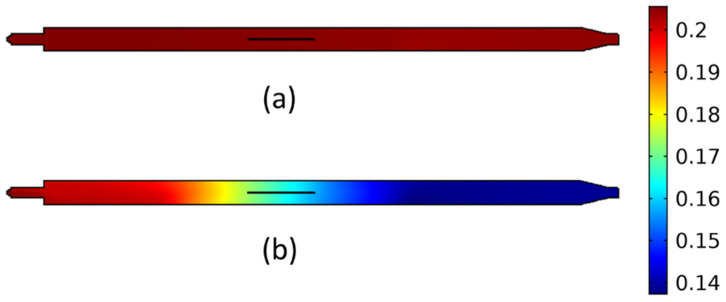
Acetylene mole fraction within the reactor for different oven temperatures: (**a**) 700 °C, (**b**) 900 °C.

The next step of the analysis focuses on the evolution of chemical species in the gas phase. The focus is primarily shifted towards acetylene, which serves as a precursor for the production of carbonaceous materials, once chemisorbed on the catalyst, and the gas-phase byproducts (C_4_H_4_), which lead to the formation of carbon impurities [20,21,31,32]. C_2_H_2_ can be chemisorbed on the catalyst surface, where it can catalytically decompose, given enough energy, leading to the formation of carbon species [20,21,29]. These species can, in turn, diffuse through the catalyst and form the carbonaceous structures, such as CNTs and CNFs [20,21,29]. On the other hand, the gaseous byproducts of acetylene decomposition lead mainly to the formation of soot and carbon impurities [31]. Therefore, during the CVD of CNTs and CNFs, a temperature window is needed, which favours the C_2_H_2_ decomposition and diffusion on the catalyst surface but, at the same time, prevents the formation of impurities by the C_2_H_2_ pyrolysis byproducts. The effect of process parameters on the evolution of the aforementioned species is crucial for the understanding of the dominating mechanisms in the gas phase, and hence, the process design for the effective deposition of CNFs and CNTs. The effect of temperature on the acetylene distribution within the CVD reactor is presented in Figure 4.

The results of Figure 4 show that the process temperature plays a crucial role on the reactive species distribution within the reactor chamber. At 700 °C, the acetylene gas-phase reactions are not activated, and thus, the acetylene mole fraction is unaffected within the chamber (Figure 4a) and equal to the mole fraction at the inlet. However, at the higher temperature of 900 °C, the gas-phase reactions are significant, leading to acetylene pyrolysis. This has a significant impact on the acetylene mole fraction, which decreases along the length of the reactor, within the heated zone. This can have a major impact on the deposition of CNTs/CNFs as less acetylene reaches the catalyst surface, restricting the acetylene chemisorption and thus the catalytic reactions, leading to the final CNT/CNF deposit. Moreover, the gas-phase reactions lead to the formation of byproducts, which can deposit carbon impurities on the catalyst surface, thus deactivating the catalyst and reducing its lifetime [20,21,31]. Finally, Figure 4b shows that at 900 °C, the acetylene mole fraction decreases along the reactor length. This means that depending on the substrate position in the reactor, the catalyst is exposed to different amounts of acetylene and gas-phase byproducts. This is another parameter that should be taken into account during the experimental design, since this will lead to non-uniform deposition along the reactor length, limiting the use of larger-area wafers.

**Figure 5:**
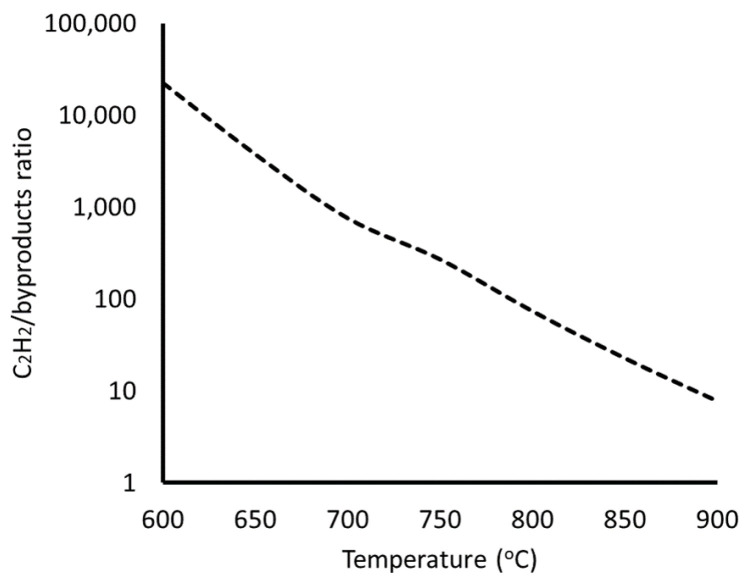
Acetylene/byproduct ratio as a function of temperature.

The above results show that the process temperature should be carefully adjusted to minimize the effects of acetylene decomposition in the gas phase to unwanted reaction byproducts. The effect of temperature on the gas composition is presented in Figure 5, where the molar ratio of acetylene and reaction byproducts on the centre of the wafer is plotted over the temperature range of 600–900 °C.

Figure 5 shows the evolution of the gas mixture composition with increasing temperature. As can be seen, minimal or no acetylene reacts at low temperature, up to 700 °C (ratio~750). At 750 °C, the ratio still has a high value (~250). However, a further increase in the temperature to 800 °C renders the gas-phase reactions significant (ratio < 100), with an acetylene-to-byproduct ratio of ~70. A further temperature increase leads to comparable concentrations of acetylene and byproducts, which can significantly affect the CNT/CNF deposition and catalyst lifetime. Such impacts of high temperature on the catalyst lifetime and the CNF/CNT quality have been previously reported in the relevant literature for CNT deposition [20,21,34,35]. As it has been shown, the catalyst lifetime is restricted at lower temperatures, due to the deposited carbon not being able to decompose and diffuse through the catalyst, thus forming a stable carbon impurity layer on the catalyst surface [20,21,35]. The increase in temperature leads to an extension of the catalyst lifetime, as the deposited carbon species diffuse within the catalyst to form CNTs. However, this occurs up to the point where the C_2_H_2_ pyrolysis is activated and the gaseous byproducts deposit on the catalyst surface as carbon impurities, thus poisoning the catalyst surface and restricting the catalyst lifetime [20,21]. Therefore, the catalyst lifetime first increases and then decreases with temperature, as shown by both computational and experimental investigations [20,21,35]. From the results so far, it is possible to deduce the optimal temperature window for the CNT/CNF deposition. This means that process temperature should be high enough to enable the catalytic decomposition of acetylene on the catalyst surface, but also ensure that the gas-phase decomposition of acetylene is not significant.

As the catalyst used in the present work is novel, it was not possible to include the surface mechanisms, and consequently, the temperature at which the catalytic reactions are activated cannot be estimated computationally, and the choice is based on previous works for the CNT deposition from acetylene on Fe catalysts, which has been found at around 650–750 °C [20,31]. From our present results, it can be seen that the effect of the gas-phase decomposition of acetylene becomes significant at temperatures above 800 °C. Therefore, the temperature range of choice for the present study would be within 700–750 °C.

##### Effect of Inlet Flow

**Figure 6:**
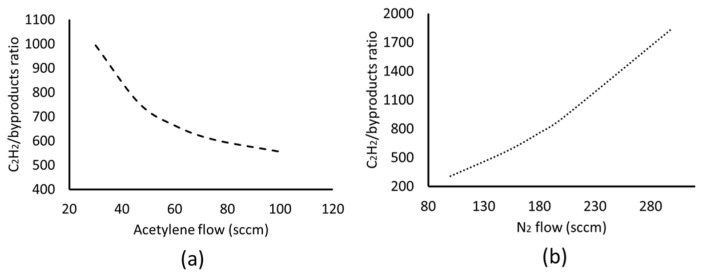
Effect of the inflow rate on the acetylene/byproduct ratio. (**a**) Varying acetylene flow with a constant N_2_ flow of 180 sccm and (**b**) varying N_2_ flow with a constant acetylene flow of 46.4 sccm.

Besides the effect of temperature, valuable insight can be obtained by the analysis of the effect of the inlet flow of the precursor and gas mixture. This effect is presented in Figure 6, where the acetylene-to-byproduct ratio at the wafer centre is plotted as a function the acetylene and N_2_ flow, for a reaction temperature equal to 700 °C.

The results of Figure 6a show that by increasing the acetylene flow, the acetylene/byproduct ratio decreases. The increase in acetylene flow increases the acetylene concentration in the reactor. Furthermore, as the acetylene pyrolysis chain starts with the reaction between two acetylene molecules [28,29], as shown by reaction 6, higher concentrations lead to higher gas-phase reaction rates and, thus, increased byproducts. Therefore, less concentrated mixtures of acetylene lead to less reaction byproducts. Similar conclusions are drawn, by the results of Figure 6b, where the increase in the N_2_ flow leads to a higher acetylene/byproduct ratio, by reducing the acetylene concentration in the reactor. Furthermore, the increase in the N_2_ carrier gas flow also increases the gas mixture velocity in the reactor chamber, reducing the residence time of the acetylene molecules in the heating zone. In this way, a smaller fraction of acetylene has the adequate residence time to react and produce byproducts.

It should be noted, however, that besides the abovementioned effects of the precursor and carrier gas flows, the optimal process design should also take into account the mechanisms and kinetics on the catalyst surface. Lower precursor concentrations may limit the formation of byproducts, but could also restrict the desired catalytic growth of CNF/CNTs. In order to take into account these phenomena, information regarding the kinetics and activation energies for the respective catalyst must be known. As the present work deals with a novel catalyst, this is not yet possible but rather the subject of a future work. In any case, even if the results of Figure 6 cannot be directly used for the optimal process design, they can provide valuable insight which, in combination with experimental measurements, may result in the desired target that is the CNFs and/or CNTs growth.

### 3.3. Deposited CNFs and CNTs on (ZrTi)Ni(SnSb)

#### 3.3.1. Visual Observation

**Figure 7:**
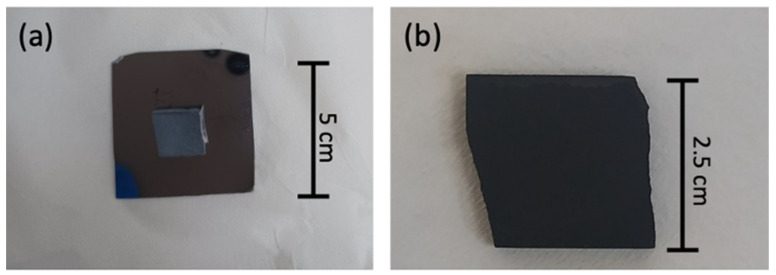
Indicative photos of the half-Heusler sample HH2 (**a**) before and (**b**) after deposition.

Figure 7 presents an indicative image of the HH2 sample before (grey, metallic square on the silicon wafer) and after deposition to illustrate the difference on the sample when carbon nanostructures are deposited, even by naked eye. A black coating has been uniformly deposited on the surface and sides of the specimen. The texture of the coating is smooth like a consolidated powder. To study the morphology of the deposited carbon, the SEM micrographs are presented in the following.

#### 3.3.2. Morphological and Structural Characterisation of CNF/CNT Coatings

**Figure 8:**
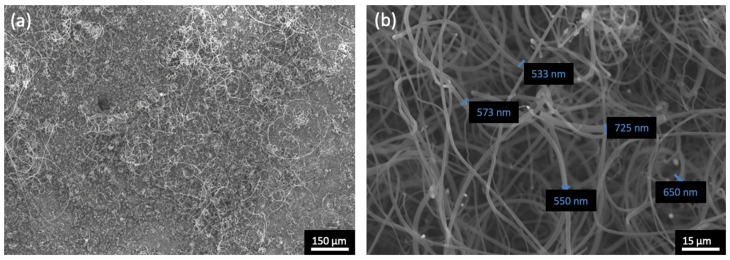
SEM micrographs for sample HH1 after deposition where carbon deposition can be observed at different areas of HH1 (**a**), and long tubular structures with thick diameters are observed (**b**), which is a magnification of (**a**).

The first half-Heusler sample, HH1, was tested under the experimental conditions shown in Table 1. After the deposition, HH1 was covered with a thick black coating similar to that shown in Figure 7, indicating that deposition occurred. For the morphological observation of the HH1 surface after deposition, SEM micrographs were observed, as presented in Figure 8. From Figure 8a, carbon deposition can be confirmed, although it seems that deposition is favoured at specific parts of the HH1 surface, i.e., there is non-uniformity existing on the coating. This can be attributed to the non-uniform elemental composition presented in Figure 1 and Table 2. The non-uniform elemental composition of the species of the half-Heusler alloy can lead to the creation of local active centres where carbon structures can grow, in the presence of Ni, and to local non-active centres, where deposition does not occur. However, by increasing the SEM magnification in areas of Figure 8a where deposition is observed (Figure 8b), tubular structures can be seen, for which the diameter ranges between 470 and 840 nm. This diameter range allows the deduction that CNFs have been formed on HH1.

**Figure 9:**
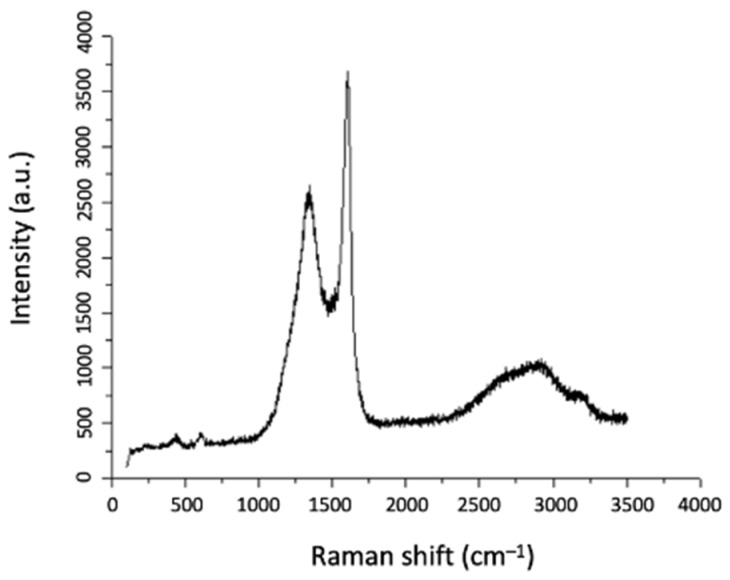
Raman spectrum of HH1 where the D, G, and 2D bands are shown at 1340 cm^−1^, 1606 cm^−1^, and 2679 cm^−1^, respectively, and the additional D+G and 2D’ bands at 2918 cm^−1^ and 3197 cm^−1^, respectively.

For further characterization of the carbon nanostructures that have been deposited, Raman spectroscopy was applied, and the spectrum obtained is presented in Figure 9. In Figure 9, the D and G bands are shown at wavelengths 1340 cm^−1^ and 1606 cm^−1^, respectively, along with the 2D band at 2679 cm^−1^. Although the D and G bands are characteristic of CNTs, the broad shape of the 2D band leads to the assumption that CNFs have been formed. Finally, the additional D+G and 2D’ bands, which are shown at 2918 cm^−1^ and 3197 cm^−1^, respectively, imply the existence of amorphous carbon in the coating, which is expected by the computational analysis, although in a small quantity.

Thus, by combining the observations from SEM micrographs of Figure 8 with the Raman spectrum of Figure 9, it can be concluded that CNFs are deposited on HH1 with co-existent amorphous carbon. The non-uniformity which is observed in Figure 8a may be attributed to the fact that the Ni, which is contained in the half-Heusler compound and is known as a catalyst for 1D carbon nanostructures [36], is not surface-oriented in the entire surface of HH1, and as a consequence, there is a locally preferential deposition on surface areas where Ni is surface-oriented. This assumption is also consistent with the evidence of Figure 1, where elemental mapping shows a non-uniform element distribution of the species of the half-Heusler compound.

**Figure 10:**
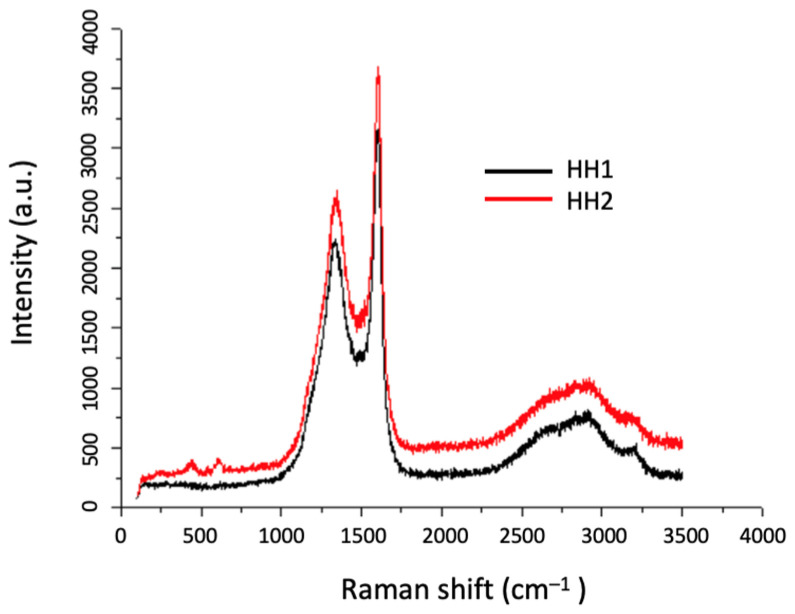
Raman spectra of HH1 (red) and HH2 (black) where it is shown that the characteristic bands of the two samples are at the same Raman shift.

Since the conditions of HH1 resulted in the formation of CNFs, sample HH2 underwent identical experimental conditions as HH1, to ensure the reproducibility of the process. The Raman spectrum obtained for HH2 is shown in Figure 10 in direct comparison with the Raman spectrum of HH1. From Figure 10, it can be seen that the characteristic bands of HH1 and HH2 are in the same Raman shift positions, and the shapes of the bands are also similar. Thus, it is possible to repeat the process applied for the deposition of CNFs on half-Heusler samples.

Upon ensuring the reproducibility of the process, the addition of H_2_ in the gas mixture was tested, targeting the enhancement in the deposited nanostructures. According to the literature, an H_2_ flow can assist in the reduction of the metallic catalyst, resulting in the better morphology of the obtained products [37]. Hence, the reactor was fed with N_2_ to ensure an inert atmosphere and H_2_ with a ratio of N_2_:H_2_ equal to 2:1, and the reduction of HH3 was conducted at 450 °C for 30 min. After this timeframe, the H_2_ flow was stopped and the deposition experiment was conducted under conditions shown in Table 1.

**Figure 11:**
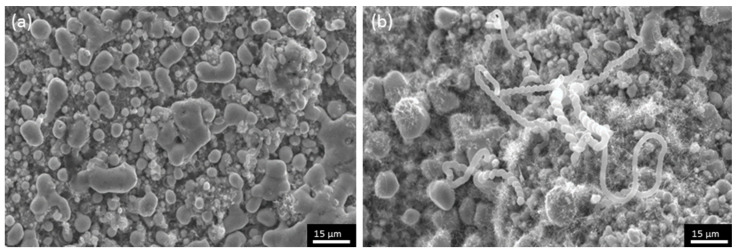
SEM micrographs of HH3 where (**a**) absent and (**b**) limited cylindrical carbon nanostructures are shown.

Figure 11 presents SEM micrographs on sample HH3. It is clearly visible that tubular structures are very limited (only evident in a spiral form) or even absent and, instead, spherical particles are observed. It can be concluded that the followed process does not lead to the formation of CNFs or CNTs. This fact can be attributed to the formation of Ni hydrides as a result of the H_2_ treatment of the sample [38,39]. The formation of nickel hydrides renders the catalytic sites of the surface inactive and, as a consequence, the targeted carbon nanostructures cannot be formed. The assumption of nickel hydride formation is strengthened by the fact that after the end of the deposition process, the sample was brittle, which is a characteristic of the hydrides under discussion. Thus, H_2_ reduction was excluded since it is shown to not be beneficial for the deposition of CNTs/CNFs on half-Heusler alloys.

**Figure 12:**
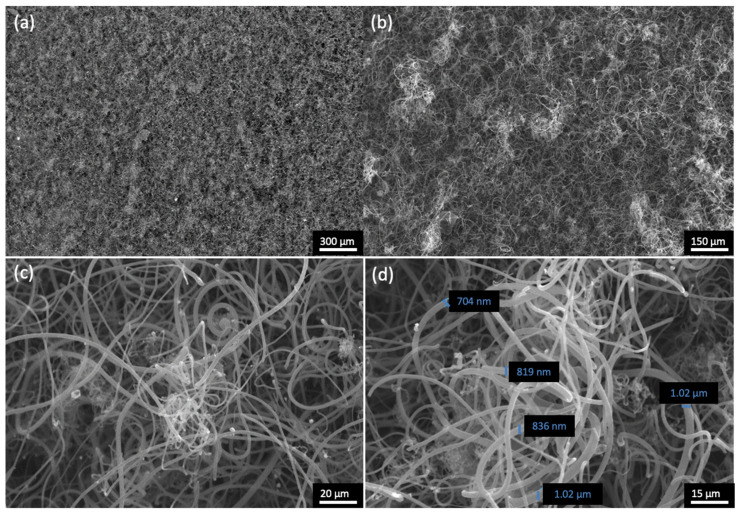
SEM micrographs of HH4 where coating uniformity is shown in (**a**,**b**) and CNFs are visible from (**c**,**d**) with the diameter size ranging between 800 and 1000 nm (**d**).

For HH4, the effect of reaction time was tested as well as the N_2_ flow, targeting the improvement in the coating uniformity. In particular, the deposition time was reduced to half and the N_2_ flow was doubled as compared to HH1 and HH2. Such an increase in the N_2_ flow can result in better a C_2_H_2_/byproduct ratio in the gas mixture that reaches the half-Heusler surface, as it has been previously shown from the computational analysis (Figure 6). In turn, this can improve the purity and uniformity of the deposited material. Additionally, thermal annealing was conducted on HH4 after the CVD experiment for 1 h, at 400 °C, and under ambient atmosphere to limit amorphous carbon which is expected to be deposited. Figure 12 presents SEM micrographs of HH4 under these reaction conditions. From Figure 12a,b, it is shown that the specific reaction conditions yielded the uniform deposition of carbon nanostructures on the surface of the half-Heusler alloy, since any aggregation and cluster from the surface is absent. Moreover, in Figure 12c,d, the formation of cylindrical nanostructures is clearly visible with the diameter ranging between 800 and 1000 nm, which corresponds to CNF formation. Also, it seems that thermal annealing has a positive effect on the deterioration of amorphous carbon.

**Figure 13:**
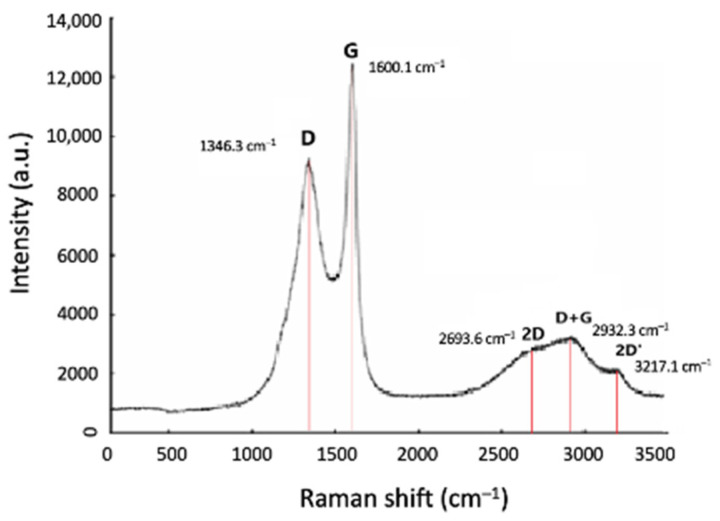
Raman spectrum of HH4 where the D, G, and 2D bands are shown at 1346 cm^−1^, 1600cm^−1^, and 2693 cm^−1^, and the additional D+G and 2D’ bands at 2932 cm^−1^ and 3217 cm^−1^, respectively.

Raman spectroscopy was also conducted with HH4 and the resulting spectrum is presented in Figure 13, where all the characteristic vibrational modes for CNFs are shown, namely the D mode at 1346 cm^−1^, the G mode at 1600 cm^−1^, and the 2D mode at 2693 cm^−1^. However, the D+G and the 2D’ modes are still shown within the Raman spectrum at 2932 cm^−1^ and 3217 cm^−1^, respectively, although from their shape, it can be qualitatively deduced that amorphous carbon is deteriorated as compared to HH1 and HH2 samples. This is consistent with what has also been pointed out from the computational model where the increase in the N_2_ flow rate results in the decrease in the deposited amorphous carbon. Thus, it can be concluded that the experimental conditions applied to HH4 lead to a uniform deposition of CNFs on the half-Heusler surface.

**Figure 14:**
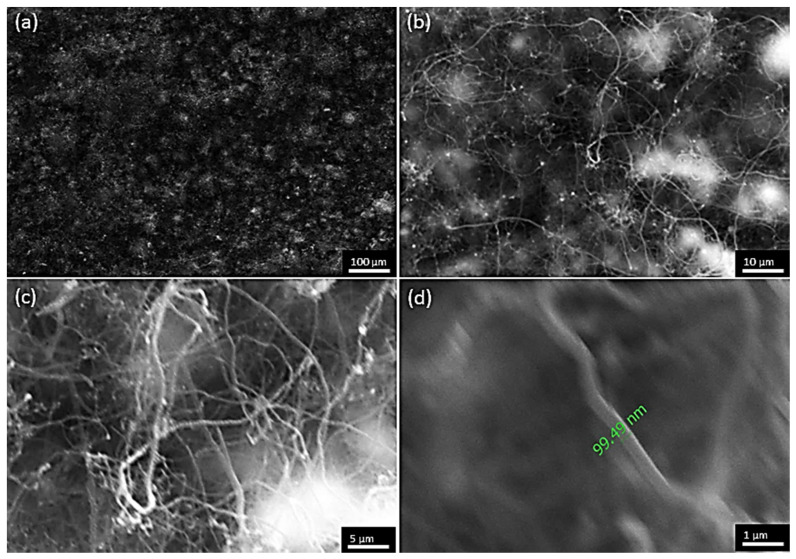
SEM micrographs of HH5 where coating uniformity is shown in (**a**,**b**), and potentially formed MWCNTs are visible from (**c**) and especially (**d**) where the diameter is of the level of 100 nm and below.

So far, CNFs have been formed on the ${\left({Zr}_{0.4}{Ti}_{0.6}\right)}_{0.33}{Ni}_{0.33}{\left({Sn}_{0.98}{Sb}_{0.02}\right)}_{0.33}$ half-Heusler compound, by applying the experimental conditions discussed above. However, towards the direction of CNT formation, it is crucial to reduce the diameter of the observed cylindrical carbon nanostructures. For this reason, for the HH5 sample, a temperature increase was attempted from 700 °C to 750 °C to check its effect on the diameter of the cylindrical carbon nanostructures. The other reaction parameters were maintained identical to those applied for HH4. This temperature increase which may result in CNT formation instead of CNFs is not part of the computational analysis, since the latter does not include any surface mechanisms and kinetics features. The results of the deposited structures at 750 °C are shown in Figure 14 in the form of SEM images. Figure 14a,b show that the temperature increase did not affect the coating uniformity, as expected from the computational analysis, since cylindrical nanostructures are shown in the entire observed surface area, especially in Figure 14b where these structures are more visible.

**Figure 15:**
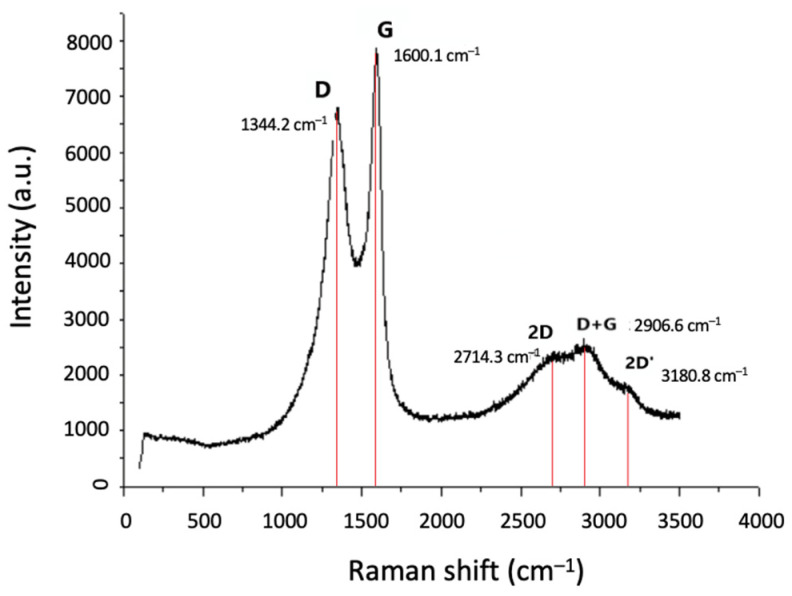
Raman spectrum of HH5 where the D, G, and 2D bands are shown at 1344 cm^−1^, 1600 cm^−1^, and 2714.3 cm^−1^, respectively, and the additional D+G and 2D’ bands at 2907 cm^−1^ and 3181 cm^−1^, respectively.

Additionally, in Figure 14c,d, the formation of these nanostructures is even more clear, although the micrographs are blurry due to the resolution limitation of the SEM device. However, in comparison with what is observed in Figure 12d for sample HH4, in this case, the diameter of the cylindrical nanostructures is within the range of 55–100 nm (indicatively, a diameter of 99.5 nm is shown in Figure 14d), which implies the formation of MWCNTs. For the further characterization of HH5, Raman spectroscopy was conducted and the spectrum is presented in Figure 15. The shape of the shown Raman modes, as well as their Raman shift, is similar to what has been shown in Figure 13. Since it is difficult to distinguish between CNFs and MWCNTs by Raman spectroscopy, further characterization is required by means of TEM to observe and confirm the formation of MWCNTs. Still, the preliminary results obtained by increasing the temperature are promising towards the possibility of depositing CNTs on half-Heusler alloys.

## 4. Conclusions

The CVD of CNFs and CNTs on the half-Heusler alloy ${\left({Zr}_{0.4}{Ti}_{0.6}\right)}_{0.33}{Ni}_{0.33}{\left({Sn}_{0.98}{Sb}_{0.02}\right)}_{0.33}$ compound was investigated for the possibility of growing carbon nanostructures directly on intermetallic compounds for the potential improvement in thermoelectric properties. The ${\left({Zr}_{0.4}{Ti}_{0.6}\right)}_{0.33}{Ni}_{0.33}{\left({Sn}_{0.98}{Sb}_{0.02}\right)}_{0.33}$ was synthesized by mechanical alloying from the constituting metals and consolidated by hot-pressing into pellets to be used in the CVD process. The XRD of the corresponding sample confirmed the formation of the desired crystal structure.

Due to the novelty of the process and the non-established experimental conditions and protocol to be applied for the CVD, a CFD model was developed for the process aiming at investigating the effect of flow and temperature conditions on the composition of the gas-phase mixture within the CVD reactor, to provide invaluable information for the design of the experimental procedure. Although theoretical predictions may deviate from reality, the validation of the applied model in a previous case study ensures the validity of the results. The model showed that temperature has a crucial effect on the distribution of species in proximity to the deposition zone; at higher temperatures, gas-phase reactions are significant, yielding the acetylene pyrolysis, which in turn impacts the deposition of CNFs/CNTs as less acetylene reaches the catalyst surface, and may lead to the formation of byproducts, which can deposit carbon impurities on the catalyst surface, deactivating the catalyst and reducing its lifetime.

By applying the computational findings in the experimental procedure and fine tuning the flow conditions according to the results of the computational analysis, we managed to deposit CNFs, with a diameter between 450 and 1000 nm, on the surface of the ${\left({Zr}_{0.4}{Ti}_{0.6}\right)}_{0.33}{Ni}_{0.33}{\left({Sn}_{0.98}{Sb}_{0.02}\right)}_{0.33}$ half-Heusler compound in a reproducible way and with high uniformity, as shown from the corresponding SEM micrographs; however, amorphous carbon has also been deposited, which could not be totally removed with thermal annealing. Furthermore, by slightly increasing the temperature from 700 °C to 750 °C, there is evidence that CNTs are deposited instead of CNFs since the observed diameters of the cylindrical structures are below 100 nm. However, for both CNFs and CNTs, further investigation and analyses are required to decide upon the carbon structures. Furthermore, investigation on the properties of the obtained samples is foreseen to test whether the current generated from the ${\left({Zr}_{0.4}{Ti}_{0.6}\right)}_{0.33}{Ni}_{0.33}{\left({Sn}_{0.98}{Sb}_{0.02}\right)}_{0.33}$ compound is better transferred due to the carbon nano-coating deposited. Still, the presented work shows the deposition of CNFs and CNTs on a half-Heusler alloy, for the first time, without the use of additional catalytic material, and presents the rationale on how the relation between computational models, experiments, and materials may lead to novel results.

Thus, further investigation is required towards the direction of depositing CNTs on the applied half-Heusler compound as well as the study of the thermoelectric properties to provide information concerning the effect of depositing carbon structures on half-Heusler compounds. The two axes will be a matter of investigation for a future publication, taking into consideration the limitations induced by the size of the half-Heusler samples, which induces challenges in measuring their thermoelectric properties.

## Figures and Tables

**Table 1:** Experimental conditions applied for the CVD of CNFs/CNTs.

ID	C_2_H_2_ Flow (mL/min)	N_2_ Flow (mL/min)	Reaction Time (min)	Reaction Temperature (°C)	Extra Steps
HH1	46.4	180	30	700	-
HH2 (repeated)	46.4	180	30	700	-
HH3	46.4	200	30	700	H_2_ flow 100 mL/min
HH4	46.4	360	15	700	Thermal annealing at 400 °C
HH5	46.4	360	15	750	-

**Table 2:** EDS analysis (semi-quantitative) on the surface of the half-Heulser alloy on three different spots, marked with coloured crosses in Figure 1.

Element	Point 1	Point 2	Point 3
C	13.8	2.9	0.0
Ti	6.6	0.4	25.1
Ni	1.4	23.7	3.6
Zr	74.0	0.0	27.1
Sn	4.2	73.0	44.2
Sb	0.0	0.0	0.0

All values represent elemental weight %.

## Data Availability

Data available on request due to restrictions (confidentiality due to running research project and trade secret on the tested materials).
